# Successful Primary Treatment of a Hydatidiform Mole with Methotrexate and EMA/CO

**DOI:** 10.1155/2009/454161

**Published:** 2009-05-19

**Authors:** M. De Vos, M. Leunen, C. Fontaine, Ph. De Sutter

**Affiliations:** ^1^Department of Gynaecology, Academic Hospital, Brussels Free University - (VUB), UZ Brussel, 1090 Brussels, Belgium; ^2^Department of Oncology, Academic Hospital, Brussels Free University - (VUB), UZ Brussel, 1090 Brussels, Belgium

## Abstract

*Background*. The preferred treatment method of most hydatidiform moles is suction aspiration. In rare circumstances uterine abnormalities may preclude surgical treatment. *Case*. We report a case of complete molar pregnancy successfully treated with methotrexate followed by EMA/CO. A 38-year-old woman with a complete hydatidiform mole and multiple uterine fibroids underwent a failed attempt at suction aspiration. Following treatment with methotrexate, a nonmetastatic persistent trophoblastic tumour developed. Six cycles of EMA/CO led to complete remission. *Conclusion*. We propose that primary treatment of molar pregnancies with chemotherapy is a useful treatment option in cases where uterine abnormalities interfere with suction aspiration.

## 1. Introduction

With an incidence of approximately 0.5 to 1 in 1000 pregnancies in Caucasians, hydatidiform mole (HM) is a relatively rare disorder of trophoblast proliferation, with broad variations in incidence worldwide [1]. HM is a pre-neoplastic disorder, and there is a substantial risk that the condition develops into a frankly malignant tumour. In that respect, it is important to distinguish between a complete hydatidiform mole (CHM), which is usually androgenetic and displays merely vestigial embryonic development, and a partial hydatidiform mole (PHM), which is typically triploid and shows coexistence of an embryo and trophoblast hyperplasia. A complete hydatidiform mole confers a greater malignant potential: 15 to 20% of CHM will eventually develop into a gestational trophoblastic tumour requiring the administration of chemotherapy, as compared to only 2 to 4% of PHM [2]. Nevertheless, irrespective of whether a hydatidiform mole is complete or partial, appropriate treatment is of uttermost importance because of the neoplastic potential. Suction curettage is regarded as the preferred treatment method [3]. Hysterectomy can be offered in selected multiparous patients but will not cure the patient if an extrauterine invasive component cannot be ruled out. Medical methods of evacuation of uterine content, such as misoprostol and antiprogesterone, are less efficient [4, 5]. In contrast with its use in low-risk nonmetastatic gestational trophoblast neoplasia (GTN) following suction curettage, there are no reports of the use of methotrexate as the primary treatment of a hydatidiform mole located within the uterine cavity. Here, we describe the management of a complete hydatidiform mole using systemic methotrexate in a patient in whom suction curettage was incomplete because of severe distortion of the uterine cavity by fibroids.

## 2. Case Presentation

A 38-year-old G_1_P_0_A_1_ was referred to the reproductive medicine outpatient clinic because of a two-year history of infertility. Her medical history was uneventful apart from menorrhagia. A transvaginal ultrasound scan revealed the existence of three intramyometrial fibroids, measuring between 30 and 40 mm. Outpatient hysteroscopy showed intrusion of the fibroids into the endometrial cavity, so that the distant half of the cavity was inaccessible for inspection with a 5.5 mm rigid hysteroscope. The patient was advized to undergo a three-month course of gonadal suppression with long-acting GnRH-agonists (triptorelin), in order to achieve a size reduction of the fibroids prior to assisted reproductive treatment. However, she declined this advice. Two months later the patient was spontaneously pregnant.

At nine-week gestation a routine ultrasound scan showed gross enlargement of the uterine cavity, hydropic changes compatible with a diagnosis of HM, and no intrauterine gestational sac. The total serum hCG level was 4282 IU/L. Suction curettage was attempted under ultrasound guidance but only yielded a very limited amount of conception products as the largest part of molar tissue was localized in the inaccessible distal part of the cavity. One day after the procedure, the hCG level dropped to 3618 IU/L. Histopathological analysis of the small tissue sample confirmed the diagnosis of a complete hydatidiform mole.

**Figure 1:**
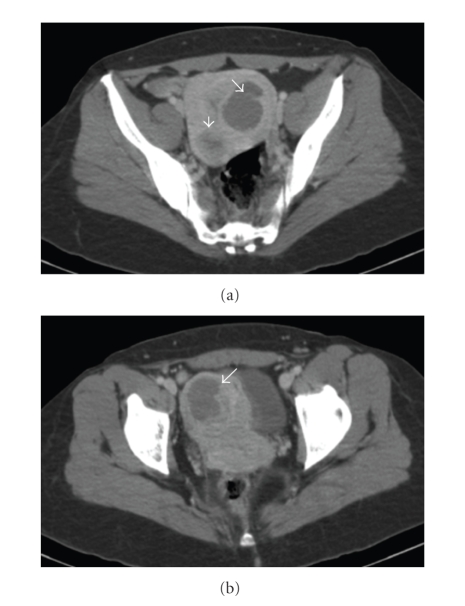
CT-scan of the abdomen, performed for GTN staging after two cycles of interval systemic methotrexate for hydatidiform mole. White arrows indicate the presence of three fibroids, resulting in important distortion of the uterine cavity. At this stage, no residual molar tissue was observed radiologically.

**Figure 2:**
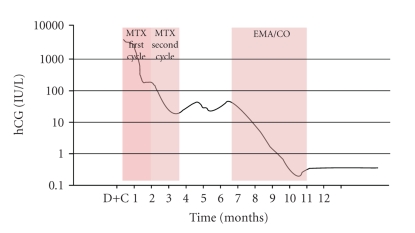
Graphical overview of hCG-levels showing initial satisfactory response (A) to two cycles of methotrexate interval, followed by a plateau-phase (B), indicating transition into a gestational trophoblastic tumour. Six cycles of EMA/CO chemotherapy were given subsequently (C).

Since the patient declined the option of a hysterectomy, interval chemotherapy with methotrexate was offered. Although there was no evidence of GTN at the start of methotrexate treatment, we adapted the interval treatment regimen for GTN, as advocated by the FIGO (FIGO 2000 management guidelines). Methotrexate 1 mg/kg was administered i.m. on days 1, 3, 5, and 7, and folinic acid 0.1 mg/kg i.m. on alternate days. This regimen was repeated after a treatment-free window of 10 days, in accordance with literature guidelines [6]. After these two cycles of methotrexate, serum hCG levels plateaued at between 10 and 40 IU/l (a total of nine fortnightly measurements), consistent with a diagnosis of GTN (Figure 2). GTN staging included a CT-scan of the thorax and of the abdomen (Figures 1(a)  and 1(b)), and did not demonstrate any extrauterine metastases. Subsequently, six cycles of EMA/CO multiagent chemotherapy, including etoposide 100 mg/m^2^, methotrexate 300 mg/m^2^ and actinomycin D 0.5 mg on day 1 and 2 and cyclophosphamide 600 mg/m^2^, and vincristine 1 mg/m^2^ (maximum dose of 2 mg) on days 8, were administered intravenously every 3 weeks. Because of paresthesia grade 3 in both feet, vincristine was omitted in the fifth and the sixth cycles. There was no evidence of hematological toxicity. Serum hCG had reached normal levels after the sixth cycle of EMA/CO. Complete remission was diagnozed after achieving over twelve months of undetectable serum hCG levels and a negative radiological evaluation.

## 3. Discussion

Reports of medical rather than surgical management of hydatidiform mole are scarce in the literature. Because of the invasive nature of trophoblast and the malignant potential of HM, optimal reduction of tumor mass is a key issue, and surgical ablative treatment (suction curettage) to evacuate the uterine content is therefore preferred above medical methods. To our knowledge, this is the first description of primary treatment of an intrauterine hydatidiform mole with methotrexate. We considered the use of methotrexate analogous to its use in nonmolar ectopic pregnancies and suggested that in general methotrexate could be considered in patients with unevacuated or incompletely evacuated molar pregnancy. Unfortunately, two cycles of methotrexate treatment in our case failed to result in complete remission of the molar disease. Although there was no evidence of distant metastases, preference was given to proceed with multiagent chemotherapy with etoposide, methotrexate, actinomycin D/cyclophosphamide, and vincristine (EMA/CO), over the single-agent actinomycin D. We are aware that single-agent with actinomycin D is indicated when the hCG value reaches a plateau below 100 IU/L, as happened in the case presented here, but we argued that the absence of a complete histological analysis of the mole, and the inherent potential of having undetected an invasive component of the tumor, justified the delivery of multiagent chemotherapy.

With this report, we aim to highlight a potential pitfall that is encountered when women of advanced reproductive age have a hydatidiform mole and present with distortion of the uterine cavity by fibroids. In our case, standard surgical treatment was not possible and nonmetastatic GTN developed following treatment with single-agent methotrexate. Nevertheless, until further experience is available, we recommend carefully monitored single-agent chemotherapy with methotrexate in the rare circumstance where uterine anomalies preclude standard surgical treatment.
